# Electrophysiological Effects of Ghrelin in the Hypothalamic Paraventricular Nucleus Neurons

**DOI:** 10.3389/fncel.2018.00275

**Published:** 2018-08-24

**Authors:** Raoni C. dos-Santos, Hanna M. Grover, Luís C. Reis, Alastair V. Ferguson, André S. Mecawi

**Affiliations:** ^1^Department of Physiological Sciences, Institute of Biological and Health Sciences, Federal Rural University of Rio de Janeiro, Seropédica, Brazil; ^2^Centre for Neuroscience Studies, Queen’s University, Kingston, ON, Canada; ^3^Department of Biophysics, Paulista School of Medicine, Federal University of São Paulo, São Paulo, Brazil

**Keywords:** corticotropin-releasing hormone, thyrotropin releasing hormone, vasopressin, oxytocin, appetite regulation, neuroendocrinology

## Abstract

The paraventricular nucleus (PVN) is involved in the control of sympathetic tone and the secretion of hormones, both functions known to be influenced by ghrelin, suggesting direct effect of ghrelin in this nucleus. However, the effects of ghrelin on the excitability of different PVN neuronal populations have not been demonstrated. This study assessed the effects of ghrelin on the activity of PVN neurons, correlating the responses to subpopulations of PVN neurons. We used a 64 multielectrode array to examine the effects of ghrelin administration on extracellular spike frequency in PVN neurons recorded in brain slices obtained from male Sprague-Dawley rats. Bath administration of 10 nM ghrelin increased (29/97, 30%) or decreased (37/97, 38%) spike frequency in PVN neurons. The GABAA and glutamate receptors antagonists abolish the decrease in spike frequency, without changes in the proportion of increases in spike frequency (23/53, 43%) induced by ghrelin. The results indicate a direct effect of ghrelin increasing PVN neurons activity and a synaptic dependent effect decreasing PVN neurons activity. The patch clamp recordings showed similar proportions of PVN neurons influenced by 10 nM ghrelin (33/95, 35% depolarized; 29/95, 30% hyperpolarized). Using electrophysiological fingerprints to identify specific subpopulations of PVN neurons we observed that the majority of pre-autonomic neurons (11/18 -61%) were depolarized by ghrelin, while both neuroendocrine (29% depolarizations, 40% hyperpolarizations), and magnocellular neurons (29% depolarizations, 21% hyperpolarizations) showed mixed responses. Finally, to correlate the electrophysiological response and the neurochemical phenotype of PVN neurons, cell cytoplasm was collected after recordings and RT-PCR performed to assess the presence of mRNA for vasopressin, oxytocin, thyrotropin (TRH) and corticotropin (CRH) releasing hormones. The single-cell RT-PCR showed that most TRH-expressing (4/5) and CRH-expressing (3/4) neurons are hyperpolarized in response to ghrelin. In conclusion, ghrelin either directly increases or indirectly decreases the activity of PVN neurons, this suggests that ghrelin acts on inhibitory PVN neurons that, in turn, decrease the activity of TRH-expressing and CRH-expressing neurons in the PVN.

## Introduction

The brain constantly monitors energy balance, changing behavior and energy expenditure as necessary to maintain metabolic status. Hunger is a sensation that arises from caloric deficit and induces food intake in order to re-establish homeostasis (Yi and Tschöp, 2012). One of the modulators of hunger is ghrelin, an orexigenic hormone secreted by the stomach during situations of caloric deficit (Müller et al., 2015). While ghrelin-induced food intake has been demonstrated in rodents and humans (Sato et al., 2012) this peptide also affects functions not directly related to food intake, and has been shown to decrease sympathetic activity (Matsumura et al., 2002; Tanida et al., 2007). In addition, intra-cerebro ventricular (ICV) ghrelin increases plasma vasopressin (AVP) (Ishizaki et al., 2002), and adrenocorticotropic hormone (ACTH) (Wren et al., 2000, 2002), and decreases thyroid-stimulating hormone (TSH) (Wren et al., 2000). These neuroendocrine effects suggest effects on corticotropin releasing hormone (CRH) and thyrotropin releasing hormone (TRH) neurons in the paraventricular nucleus (PVN) of the hypothalamus. *In vitro*, ghrelin induces AVP and CRH release in hypothalamic explants (Wren et al., 2002; Mozid et al., 2003), and oxytocin (OT) and AVP release in neurohypophyseal cell culture (Gálfi et al., 2016). Food deprivation, which increases ghrelin, reduces TRH (Mori et al., 1988; Blake et al., 1991), and TRH-mRNA levels in the brain decrease after peripheral ghrelin injection, suggesting that TRH neurons are affected by ghrelin (Pekary and Sattin, 2012).

The effects of ghrelin on blood hormone levels and autonomic regulation indicate the PVN as a target nucleus where central ghrelin may act to elicit these effects. The PVN is composed of magnocellular and parvocellular neurons. Magnocellular neurons project to the neurohypophysis where, on depolarization, they release AVP and OT into the systemic circulation. Parvocellular neuroendocrine neurons secrete CRH and TRH into the hypophyseal portal circulation at the median eminence which act in the adenohypophysis to cause release of ACTH and thyrotropin into the general circulation (Stern, 2015). Pre-autonomic parvocellular neurons projecting to the brainstem and spinal cord play critical roles in the regulation of autonomic systems (Stern, 2001) while those that project to other brain regions mediate diverse physiological processes (Knobloch et al., 2012). Thus, the PVN controls sympathetic tone and the secretion of hormones, both functions known to be influenced by ghrelin, suggesting direct effect of ghrelin in this nucleus. In accordance with this hypothesis the growth hormone secretagogue receptor (GHSR), the only described ghrelin receptor (Kojima et al., 1999), is present in the PVN (Cowley et al., 2003; Harrold et al., 2008). In addition, ICV ghrelin binds in the PVN (Cabral et al., 2013) and both ICV (Lawrence et al., 2002; Olszewski et al., 2007; Cabral et al., 2012; Stevanovic et al., 2014) and peripheral (Rüter et al., 2003; Kobelt et al., 2008; Cabral et al., 2012) injections of ghrelin increase the activity of PVN neurons while intra-PVN injection of ghrelin induces feeding (Wren et al., 2001; Melis et al., 2002; Olszewski et al., 2003; Shrestha et al., 2004). Electrophysiological studies have shown that ghrelin reduces inhibitory (Cowley et al., 2003) and excitatory (Kola et al., 2008) post-synaptic currents in the PVN. It has also been suggested that ghrelin inhibits γ-aminobutyric acid (GABA) releasing PVN neurons, in turn increasing the activity of CRH PVN neurons, an effect that is independent of the arcuate nucleus (ARC) (Cabral et al., 2016).

Taken together, these observations suggest that the PVN mediates, at least some of the central effects of ghrelin, however, the effects of ghrelin on the excitability of different PVN neuronal populations have not been demonstrated. We hypothesized that ghrelin would influence the activity of PVN neurons, and may exert different effects on different subpopulations of neurons within this hypothalamic nucleus.

## Materials and Methods

### Ethical Approval

All animal protocols were approved by the Queen’s University Animal Care Committee, conformed to the standards of the Canadian Council on Animal Care and were in accordance with the “Guide for the Care and Use of Laboratory Animals: Eighth Edition, NIH, 2011.”

### Animals

We used 25–30 days old Male Sprague-Dawley rats (Charles River, Quebec, Canada) (50–100 g) for all experiments. Since healthy viable neurons are harder to obtain from brain slices taken from adult animals, our lab (Loewen and Ferguson, 2017) and others (Luther and Tasker, 2000; Ma and Liu, 2012) have previously used juvenile rats to measure the effects of peptides on PVN neurons. However, brain functions may differ between juvenile and adult rats, thus, the age of the animals is a methodological limitation of the study. Animals were housed in a room maintained at 22°C under a 12:12 h light-dark cycle with food and water *ad libitum*. A total of 53 rats were used in these experiments from which hypothalamic brain slices were obtained either for extracellular recordings (9 slices, obtained from 4 rats), or patch clamp (49 rats – 122 neurons, 45 of which were used for RT-PCR analysis). Anesthesia influences PVN neuronal activity (Tanaka et al., 1989) and AVP release (Reed et al., 2009), therefore decapitation was carried out in non-anesthetized animal.

### Electrophysiology

Rats were decapitated and the brains were removed and immersed in ice-cold slicing solution (87 NaCl, 2.5 KCl, 25 NaHCO_3_, 0.5 CaCl_2_, 7 MgCl_2_, 1.25 NaH_2_PO_4_, 25 glucose and 75 sucrose, in mM) and continuously aerated with 95% O_2_/5% CO_2_. The brain was blocked and 300 μm coronal slices containing the PVN were cut using a vibratome (VT1000 S; Leica, Nussloch, Germany). Slices were then incubated at 32°C for at least 1 h in artificial cerebrospinal fluid (aCSF) (124 NaCl, 2.5 KCl, 20 NaHCO_3_, 2 CaCl_2_, 1.3 MgSO_4_, 1.24 KH_2_PO_4_ and 10 glucose, in mM), after which they were used for extracellular or intracellular recordings.

### Extracellular Recordings

**FIGURE 1:**
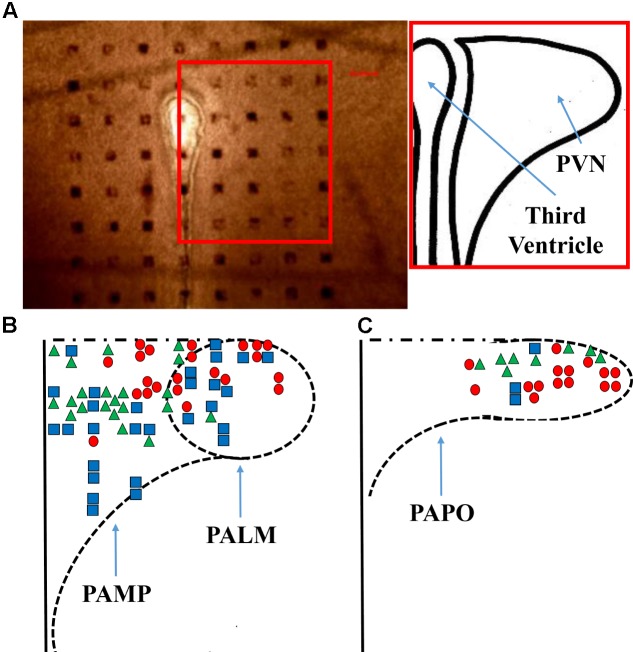
Probe positioning. **(A)** A representative micrograph of the PVN slice positioning over the probe is shown, the bar indicates 100 μm and the inset shows a representation of the nucleus. A diagram **(B,C)** shows all the responses obtained from the application of ghrelin. PAMP = medial parvocellular; PALM = lateral magnocellular; and PAPO = posterior parvocellular portions of the paraventricular nucleus. Triangles, squares, and circles represent, respectively, increased, unchanged, and decreased firing frequency.

Extracellular recordings were performed on planar-multielectrode array (MED-64 system; Alpha MED Sciences, Osaka, Japan). An 1 mm 8 × 8 electrode arrangement, with 50 μm × 50 μm electrodes and a 150 μm distance between electrodes was used. Signals were recorded at a sampling rate of 20 kHz and 16-bit resolution. Slices were placed on the probe, submersed in carbogenated aCSF and held in place by a small weight with a net. The correct placement of the slice was assessed on a microscope, a representation of the PVN placement over the probe is shown (**Figure 1**). Then the probe was connected to the MED64 system, in which a flow of 1–2 ml/min of carbogenated aCSF, heated to 35°C was maintained. After a stabilization period of 10 min, baseline activity was recorded for at least 5 min. Then, 10 nM ghrelin, diluted in aCSF, was applied for 2 min and recording proceeded for an additional 30 min. Channels positioned over the PVN that showed activity during the experiment were analyzed. Mobius software (Alpha MED Sciences, Osaka, Japan) was used to automatically sort spike shapes and identify neurons within each channel, the threshold of detection was set above noise levels for each channel (ranging from |10| to |13| μV) and spikes with a similarity greater than 60% that appeared a minimum of 100 times were considered. Spike frequency for individual neurons was plotted in bins of 30 s. 4 min immediately before ghrelin hits the bath and 4 min of peak effect were compared and neurons were classified as affected by ghrelin when application changed firing rate by more than ± 20% of mean baseline (Yousheng et al., 2008).

In order to assess whether ghrelin effects were direct or dependent on prior activation of glutamatergic or GABAergic neurons, the effects of ghrelin were assessed in the presence of 10 μM bicuculline methiodide and kynurenic acid, respectively, GABAA and glutamate receptor antagonists (Latchford and Ferguson, 2004). After 10 min of stabilization, baseline was recorded for 5 min after which aCSF was changed to aCSF with antagonists. After 5 min, ghrelin 10 nM diluted in aCSF with antagonists, was applied for 2 min and the responses were analyzed as previously described.

### Intracellular Recordings

Patch electrodes were manufactured with borosilicate glass (World Precision Instruments, Sarasota, FL, United States) pulled by a micropipette puller (P-97; Sutter Instrument, Novato, CA, United States) and filled with intracellular solution (125 potassium gluconate, 10 KCl, 2 MgCl2, 0.1 CaCl2, 5.5 EGTA, 10 HEPES, 2 NaATP, and adjusted to pH 7.2 with KOH, in mM). Micropipettes with resistances between 3 and 5 MΩ were used. Slices containing the PVN were transferred to a recording chamber continuously perfused with carbogenated aCSF, heated to 35°C at flow between 1.5 and 2 ml/min and used for whole-cell current clamp recording. Neurons were visualized using a 40× water immersion objective mounted on an upright microscope with differential interference contrast optics (Scientifica, East Sussex, United Kingdom). A seal of at least 1 GΩ resistance was achieved, then brief suction was applied to rupture the membrane and obtain whole cell access. Whole cell recordings were obtained using a MultiClamp 700B amplifier (Molecular Devices, Sunnyvale, CA, United States), with a sampling rate of 10 kHz, filtered at 2.4 kHz, using a Micro1401 mk II interface and Spike2 software (Cambridge Electronic Design, Cambridge, United Kingdom) for offline analysis. Neurons were classified in accordance with membrane potential responses to hyperpolarizing pulse protocols. Neurons with a large A-current were classified as magnocellular (Tasker and Dudek, 1991), neurons with low-threshold calcium spikes were classified as pre-autonomic (Stern, 2001) and neurons with neither of these characteristics were classified as neuroendocrine (Luther et al., 2002). Following a minimum 5 min stable baseline recording of membrane potential ghrelin diluted in aCSF was applied to the slices for 2 min and the responses were recorded until a return to baseline or loss of GΩ seal. Effects of ghrelin were assessed by comparison of the mean membrane potential during the 100 s immediately prior to ghrelin application with the 100 s of peak effect within 10 the minutes following peptide application. The effects of ghrelin on membrane potential were considered significant if the change was greater than 2 mV and two times baseline SD. A liquid junction potential of 15 mV was subtracted from all reported membrane potentials.

### Single Cell RT-PCR

Single cell RT-PCR was carried out as previously described (Pires da Silva et al., 2016). In short, immediately after electrophysiological recording the content of the micropipette was collected and used for cDNA generation with High Capacity cDNA Reverse transcription kit (Invitrogen, Carlsbad, CA, United States), followed by a pre-amplification step with TaqMan Pre-Amp Master Mix Kit (Invitrogen, Carlsbad, CA, United States), and the probes for vasopressin (Rn00566449_m1), oxytocin (Rn00564446_g1), CRH (Rn01462137_m1), TRH (Rn00564880_m1), and β-Actin (Rn00667869_m1). Then, RT-PCR was performed in simplex and triplicates, using the aforementioned probes and TaqMan Universal PCR Master Mix kit (Invitrogen, Carlsbad, CA, United States). All reactions were carried out according to manufacturers recommendations and β-Actin was used as an endogenous control. Samples in which β-Actin was not present were removed from the analysis and the remainder were classified by the presence of mRNA for the target genes. The mRNA was considered as present when the amplification threshold occurred before cycle 36.

### Chemicals and Drugs

All solutions were prepared on the day of the experiment. Ghrelin was purchased from Phoenix Pharmaceuticals (Belmont, CA, United States); all RT-PCR reagents were purchased from Applied Biosystems (Foster City, CA, United States); bicuculline methiodide, kynurenic acid and all salts used for the preparation of aCSF, slicing solution and intracellular solution were purchased from Sigma Pharmaceuticals (Oakville, ON, Canada).

### Statistical Analysis

GraphPad Prism v. 7.0 was used for all statistical analyses. Proportions were compared with Chi-Square test. Magnitudes were compared with one-way analysis of variance followed by Tukey’s *post hoc* test. The duration of effects was compared with unpaired *t*-test or one-way analysis of variance where appropriate. The proportional changes in firing frequency were compared with two-way analysis of variance followed by Sidak *post hoc* test. Statistical significance was set at *p* < 0.05 and all data is described as mean ± standard deviation.

## Results

**FIGURE 2:**
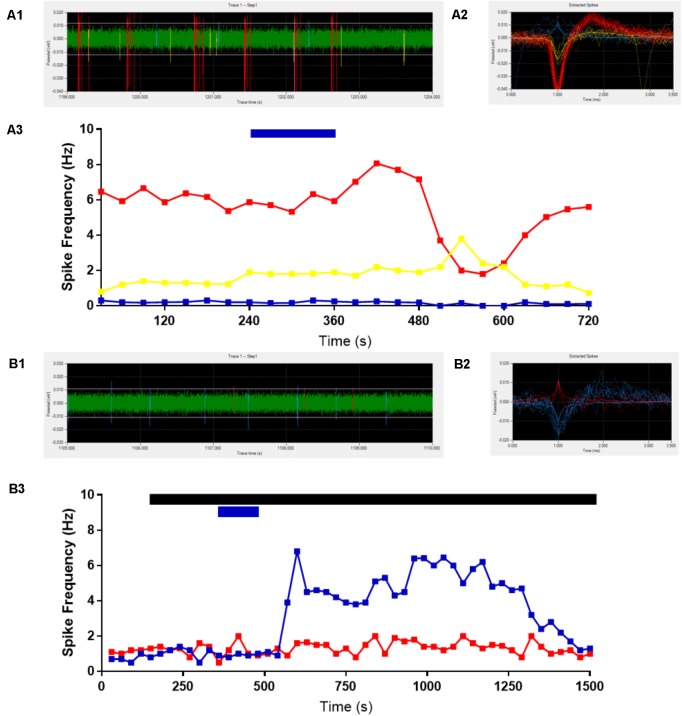
Representative extracellular recordings. Channels with detectable spikes and correct positioning were selected for analysis. One channel is shown as representative **(A)**, 3 neurons were identified **(A1,A2)** and ghrelin application decreased (red line), increased (yellow line) or did not change (blue line) firing frequency (1/3 each) **(A3)**. The addition of GABAA and Glutamate receptor antagonists to the bath (black bar) abolishes the decreases in activity due to 10 nM ghrelin application (blue bar). Another representative recording is shown **(B)**, in which two neurons were identified in the trace **(B1,B2)**. Ghrelin increased firing frequency in one (blue line) and did not affect the other (red line) **(B3)**.

**FIGURE 3:**
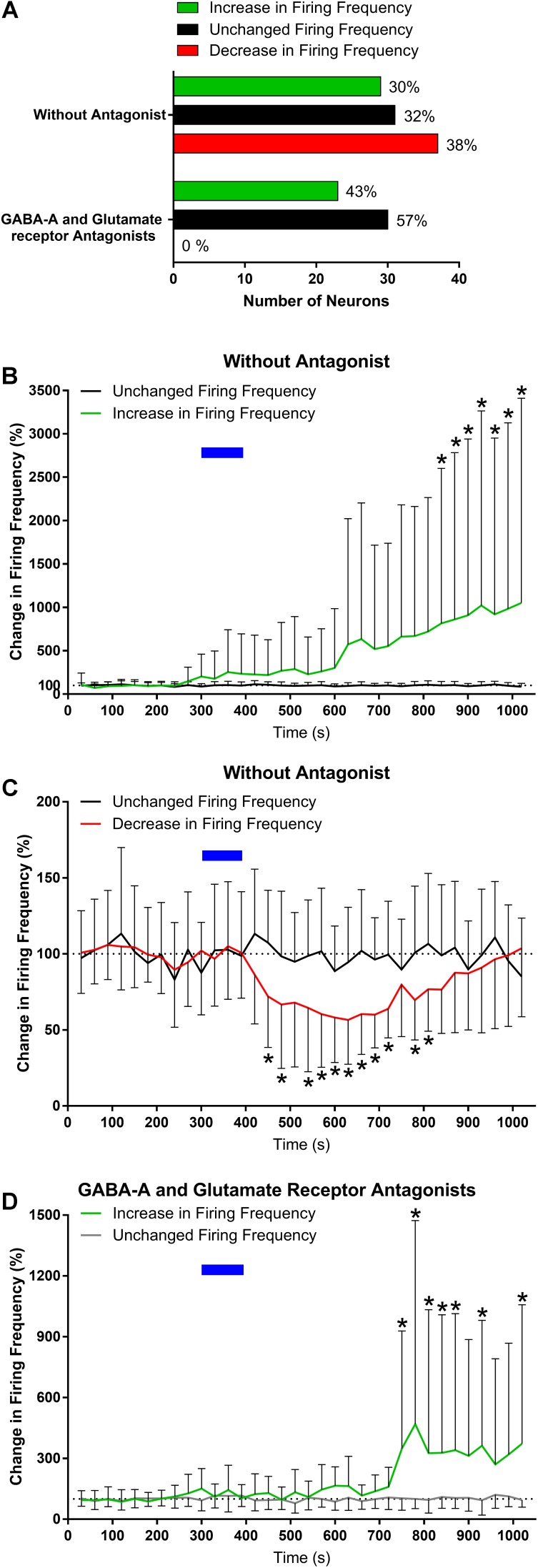
Ghrelin affects the firing frequency of PVN neurons. Summary data from the extracellular recordings demonstrates that ghrelin affects most PVN neurons, either increasing or decreasing firing frequency; and that with the addition of glutamate and GABAA antagonists the decreases in activity are not present **(A)**. The proportion of change was significantly different in both the increases **(B)** and decreases **(C)** in firing frequency when compared to the group with unchanged firing frequency. Ghrelin effects were also significantly different in the presence of GABAA and glutamate receptor antagonists **(D)**. ^∗^*p* < 0.05 vs. unchanged firing frequency.

**FIGURE 4:**
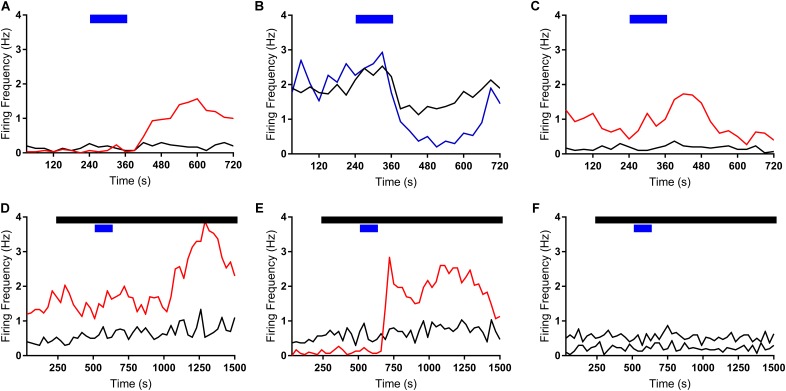
Ghrelin effects vary within individual neurons. Additional representative recordings are shown, where each rate meter record **(A–F)** represents all data acquired from one specific channel and each line represents a single neuron. Bath application of ghrelin incurs both increases (red lines) and decreases (blue lines) in firing frequency. Black bar indicates application of GABAA and glutamate receptor antagonists; Blue Bar indicates ghrelin application.

We first assessed the effects of ghrelin on extracellular spike frequency in PVN neurons in extracellular recordings obtained from 10 different hypothalamic slices. Only channels in which spikes were detectable and the PVN was correctly placed above the probes were analyzed. Channels outside of the PVN seldom showed spikes. A representative analysis (**Figure 2**) shows examples of 1 channel in one specific recording where extracellular spikes were detectable (**Figure 2A**); the detection threshold was set above noise values individually for each channel (**Figure 2A1**). The software then detects individual neurons based on the shape of the spikes (**Figure 2A2**). The firing frequency for each neuron was plotted in 30 s bins and the response to 10 nM ghrelin was observed (**Figure 2A3**). In the representative channel (**Figure 2A**), 3 neurons were recorded of which 1 neuron increased, 1 decreased, and 1 showed no change in spike frequency. In total (**Figure 3A**), 48 channels detected spikes in 7 slices, in which 1–3 neurons/channel were identified. A total of 97 neurons were recorded, of which 29 (30%) increased firing frequency and 37 (38%) decreased firing frequency in response to bath administration of 10 nM ghrelin. In a second series of recordings we assessed whether the responses to ghrelin were dependent on the modulation of glutamatergic or GABAergic neurotransmission by examining the effects of ghrelin in the presence of 10 μM bicuculline methiodide and kynurenic acid. In these recordings 27 channels detected spikes in 2 slices, in which 1–2 neurons/channel were identified, as illustrated in **Figure 2B**, single channels recorded neurons which were either depolarized or unaffected, but no cells in which spike frequency was inhibited (**Figure 2B3**). A total of 53 neurons were recorded (**Figure 3A**), of which 23 (43%) increased firing frequency and 30 (57%) were not affected by bath administration of ghrelin. These proportions of neurons responding to ghrelin were significantly different in the presence of glutamate and GABAA antagonists (Chi-square = 27.14, df = 2, *p* < 0.001). This indicates that GABAA and/or glutamate receptors activation is necessary for ghrelin to decrease the activity of PVN neurons. Additional representative channels showing the response to ghrelin in the absence (**Figures 4A–C**) or presence (**Figures 4D–F**) of GABAA and glutamate receptor antagonists demonstrate that individual neurons respond differently to ghrelin application. We hypothesize that these differences in response are due to the different subpopulations of neurons in this nucleus. In the neurons measured without the antagonists, the proportion of changes in firing frequency demonstrated a significant difference between neurons that increased firing frequency and neurons in which firing frequency was unchanged [*F*_(33,1881)_ = 4.27, *p* < 0.001, **Figure 3B**], and between neurons in which the firing frequency decreased and neurons that were unaffected [*F*_(33,2178)_ = 4.44, *p* < 0.001, **Figure 3C**]. Similarly, in neurons evaluated in the presence of antagonists, neurons that increased in firing frequency were significantly different from unaffected neurons [*F*_(33,1683)_ = 3.45, *p* < 0.001, **Figure 3D**].

**FIGURE 5:**
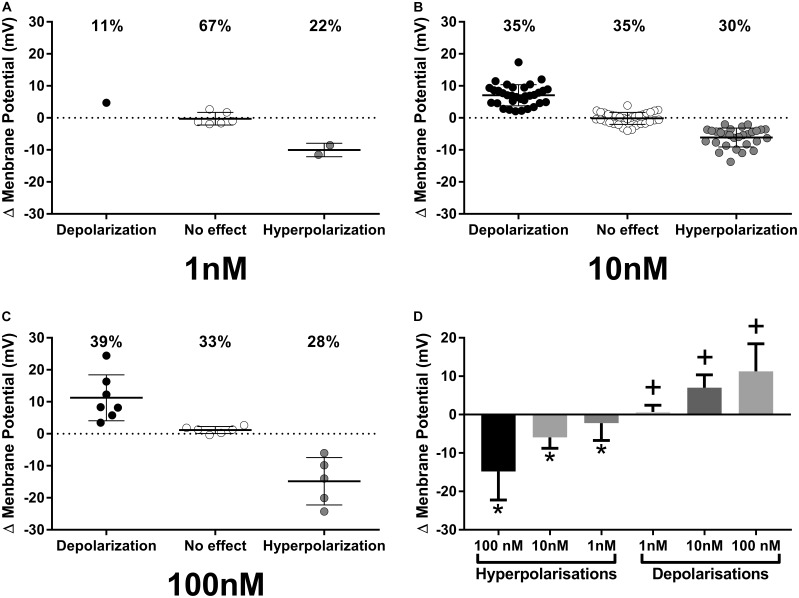
Intracellular recordings of PVN neurons responses to ghrelin. Proportion and magnitude of PVN neurons responses to ghrelin in 1 **(A)**, 10 **(B)** and 100 **(C)** nM concentrations; Concentration-response of ghrelin effects **(D)**. Ghrelin affects PVN neurons in all concentrations tested and the effects are concentration-dependent. ^∗^*p* < 0.05 against other hyperpolarizations; ^+^*p* < 0.05 against other depolarizations.

While our extracellular recordings clearly demonstrate effects of ghrelin on spike frequency in PVN neurons, these data provide no direct insight as to either, the cellular mechanisms of action on single PVN neurons, or the specific subpopulations of PVN neurons affected. We therefore utilized whole-cell patch clamp recording techniques to examine specific effects of ghrelin on single PVN neurons. Bath administration of varying concentrations of ghrelin (1, 10, and 100 nM) also affected PVN neurons (**Figure 5**, 1 nM: 11% depolarized, 22% hyperpolarized, *n* = 9; 10 nM: 35% depolarized, 30% hyperpolarized, *n* = 94; 100 nM: 39% depolarized, 28% hyperpolarized, *n* = 18). Statistical analysis of the changes in membrane potential showed that both depolarizations [*F*_(2,44)_ = 12.89, *p* < 0.001, ONE-WAY ANOVA] and hyperpolarizations [*F*_(2,39)_ = 16.81, *p* < 0.001, ONE-WAY ANOVA] were significantly different between ghrelin concentrations; Tukey’s *post hoc* analysis showed that all concentrations effects on membrane potential were significantly different between each other (*p* < 0.05). Therefore, although the proportion of ghrelin responsive neurons were similar in 10 and 100 nM (respectively, 65 and 67%), the magnitude of response is concentration-dependent. Notably, the proportion of responses to 10 nM ghrelin were not significantly different between intracellular and extracellular recordings (Chi-square = 1,27, df = 2, *p* = 0.53).

In regards to the duration of effects, 1 and 10 nM ghrelin induced similar responses (1 nM = 434 ± 250 s vs. 10 nM = 572 ± 312 s, *p* = 0.45, unpaired *t*-test). Administration of 100 nM ghrelin induced a long-lasting response that persisted until the seal was broken in 50% of ghrelin-responsive neurons (6/12). The remaining neurons showed a large variability in the duration of response, with two neurons in which effects lasted for a longer period (respectively, 1916 and 4024 s) and four that showed effects similar to the other concentrations [499.8 ± 126.7 s; *F*(2,65) = 1.844, *p* = 0.16, ONE-WAY ANOVA]. Thus, the 100 nM concentration extends the effects of ghrelin on most PVN neurons, whilst 10 and 1 nM cause effects of similar duration. Further analyzing 10 nM ghrelin, no significant differences were found between hyperpolarizing and depolarizing ghrelin-responsive neurons (Depolarizing = 572.7 ± 357.2 vs. hyperpolarizing = 571.8 ± 252.8, *p* = 0.99, unpaired *t*-test) or between different neuronal phenotypes [Neuroendocrine = 576.4 ± 268.6 s; pre-autonomic = 601.6 ± 438.6 s; magnocellular = 532.8 ± 271.3 s; *F*(2,59) = 1.225, *p* = 0.30, ONE-WAY ANOVA]. These results suggest that, within the same concentration, the duration of ghrelin effects are consistent in PVN neurons.

**FIGURE 6:**
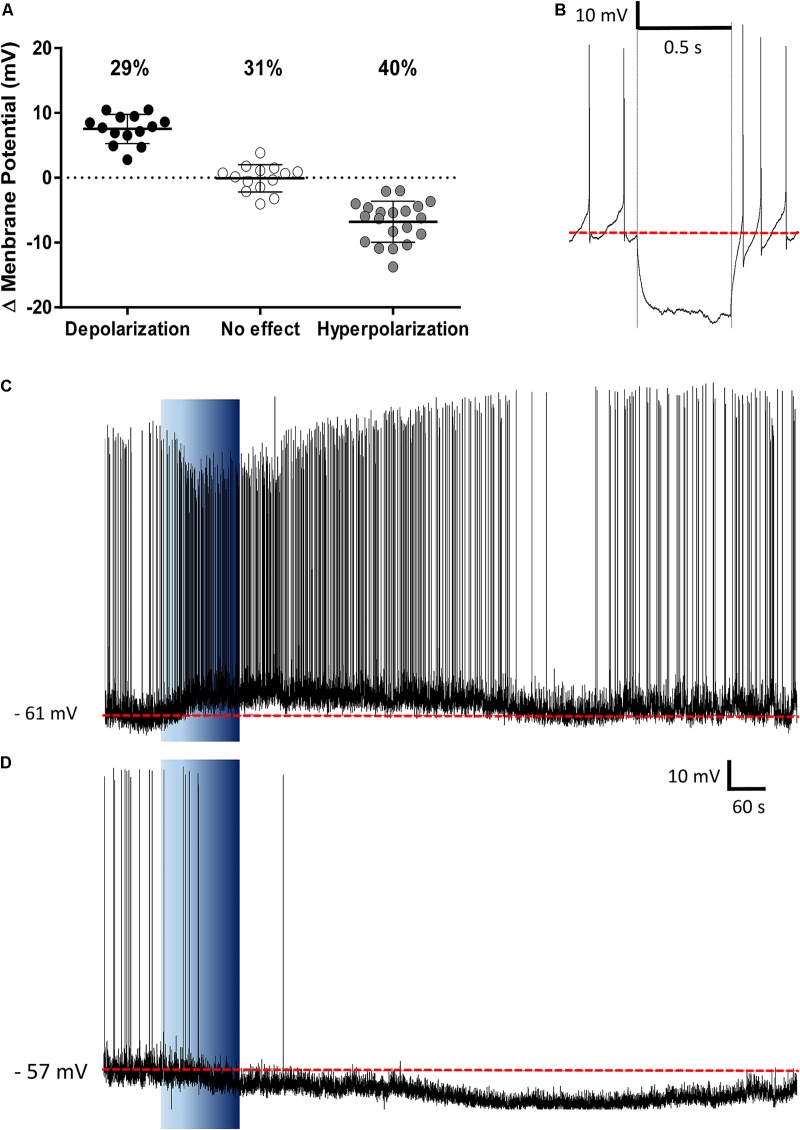
Ghrelin affects neuroendocrine PVN neurons. Ghrelin changes membrane potential in neuroendocrine PVN neurons **(A)**, with populations of neurons either increasing or decreasing membrane potential in response to ghrelin. Neuroendocrine neurons were identified by the lack of an electrophysiological fingerprint in response to a hyperpolarizing pulse **(B)**. Representative traces of a depolarization **(C)** and a hyperpolarization **(D)** are shown. Background indicates 10 nM ghrelin application to the bath, dashed line represents mean baseline membrane potential.

**FIGURE 7:**
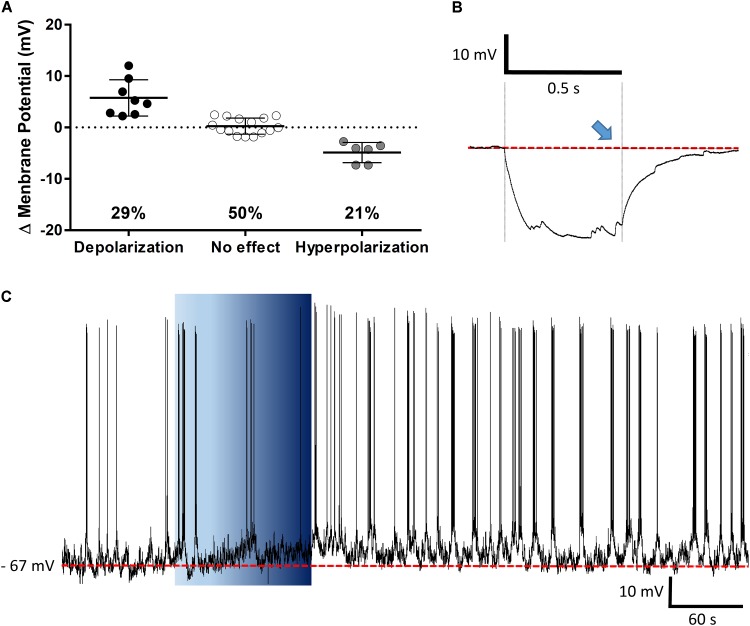
Ghrelin affects magnocellular PVN neurons. Ghrelin does not affect half of PVN magnocellular neurons, while some neurons either depolarize or hyperpolarize in response to ghrelin **(A)**. Neurons were defined as magnocellular due to the presence of A-current, characterized by the delay to return to baseline (blue arrow) after a hyperpolarizing pulse **(B)**, a representative trace of a magnocellular neuron that increases membrane potential and firing frequency after ghrelin application is shown **(C)**. Background indicates 10 nM ghrelin application to the bath, dashed line represents mean baseline membrane potential.

**FIGURE 8:**
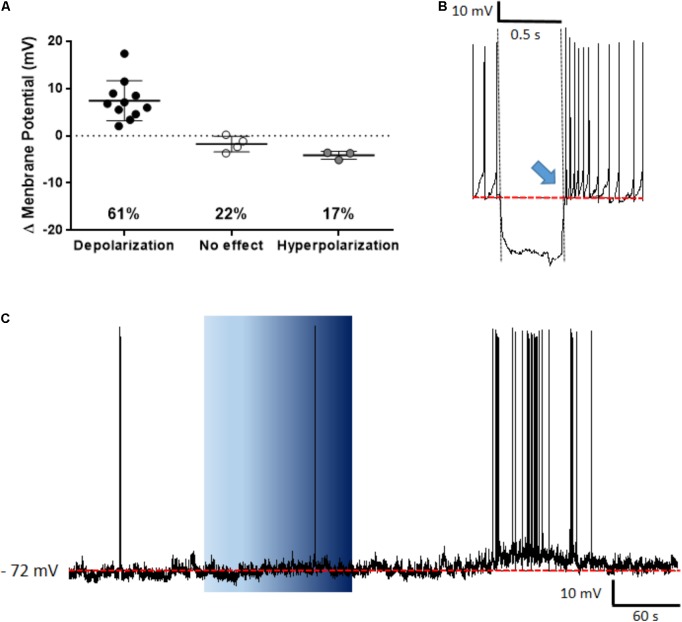
Ghrelin affects pre-autonomic PVN neurons. Ghrelin depolarizes most pre-autonomic PVN neurons **(A)**. Neurons were defined as pre-autonomic due to the presence of low-threshold spikes (blue arrow) after a hyperpolarizing pulse **(B)**, a representative trace of a depolarization is shown **(C)**. Background indicates 10 nM ghrelin application to the bath, dashed line represents mean baseline membrane potential.

The PVN showed populations of neurons that depolarized, hyperpolarized or were unaffected by ghrelin and we speculated that these effects might be distributed to different functional subpopulations of PVN neurons. In order to assess if these differences in response were dependent on the neuronal phenotype, cells were sorted in accordance to the electrophysiological response to a hyperpolarizing pulse, as previously described (Tasker and Dudek, 1991; Stern, 2001; Luther et al., 2002). While significant proportions of neuroendocrine (29% depolarizations, 40% hyperpolarizations; **Figure 6**), and magnocellular neurons (29% depolarizations, 21% hyperpolarizations; **Figure 7**) responded to ghrelin, mixed effects were still observed in both groups of cells. In contrast, the majority of pre-autonomic neurons tested depolarized to ghrelin (61% depolarizations, 17% hyperpolarizations; **Figure 8**) suggesting primarily excitatory effects on these cells.

**FIGURE 9:**
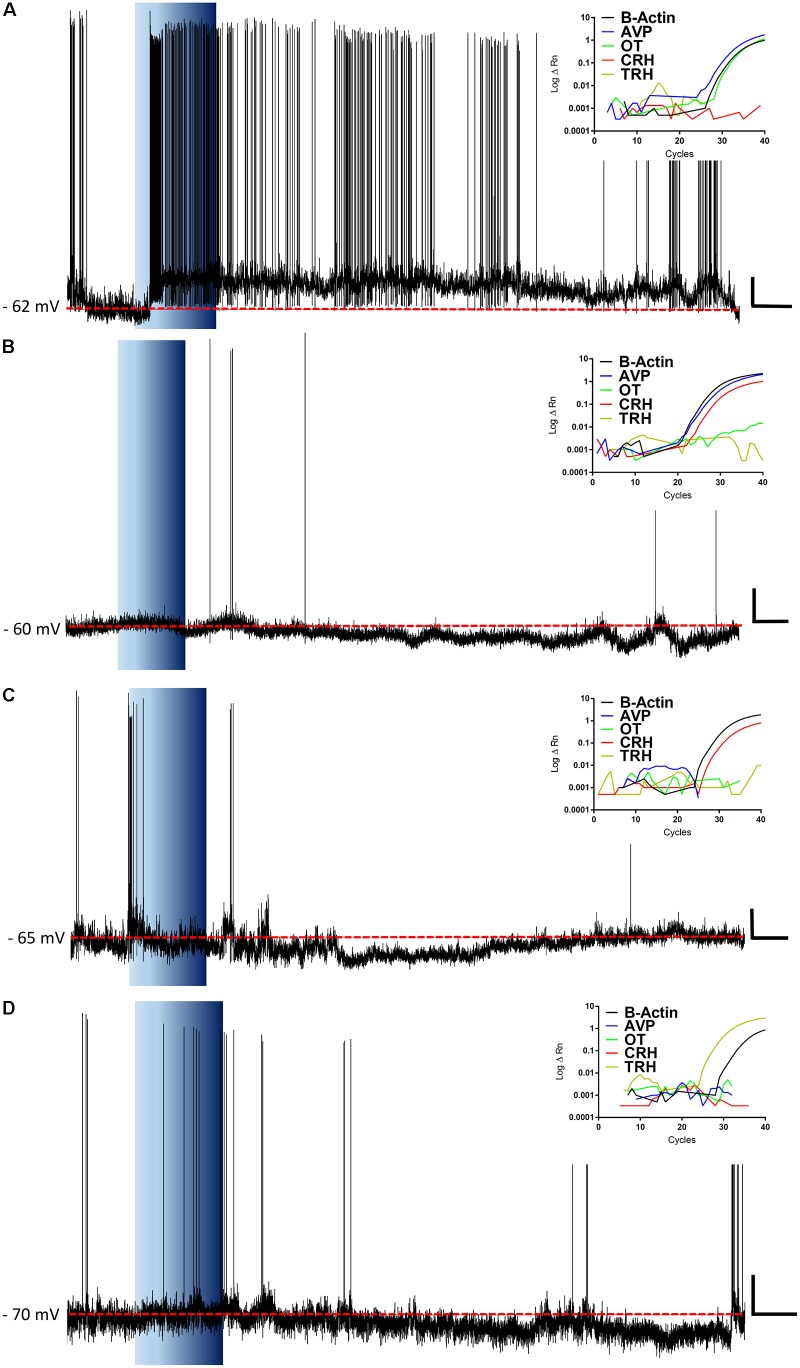
Representative recordings and RT-PCRs. The figure shows representative traces for ghrelin responsive neurons and the insets show their respective amplification plots for β-Actin, AVP, OT, CRH, and TRH mRNA. Note, a neuron that depolarizes in response to ghrelin and expresses both AVP and OT mRNA, with the predominance of AVP **(A)**; hyperpolarizations in CRH expressing neurons, either with AVP-CRH coexpression **(B)** or with CRH only **(C)**; and a hyperpolarization in a TRH neuron **(D)**. Blue background indicates 10 nM ghrelin application; red dashed line represents mean membrane potential; vertical scale bar represents 10 mV; and horizontal scale bar represents 60 s.

**FIGURE 10:**
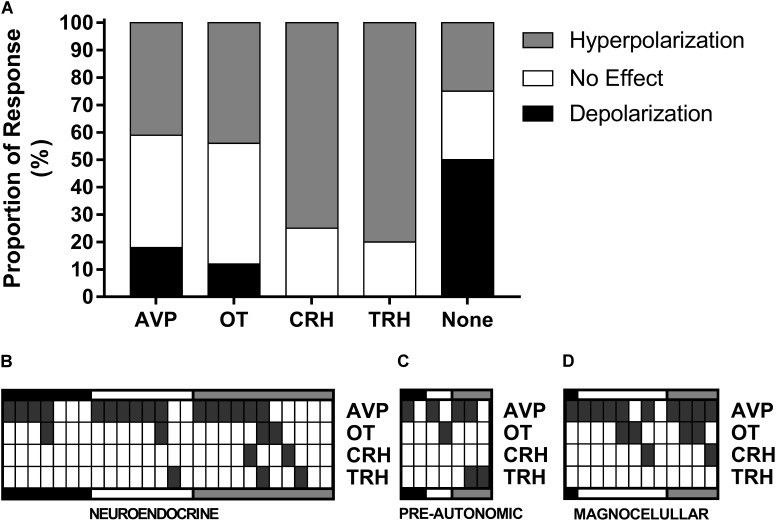
Single-cell RT-PCR. The proportions of responses to ghrelin for each gene **(A)** showed that most TRH and CRH neurons hyperpolarize in response to ghrelin and most depolarizations come from neurons with none of the tested genes. Individual data from neuroendocrine **(B)**, pre-autonomic **(C)**, and magnocellular **(D)** neurons shows that AVP mRNA is expressed in most neurons and that the majority of CRH neurons co-express AVP.

These data demonstrate, that even within these electrophysiologically defined subgroups within which different chemical phenotypes exist (e.g., magnocellular OT and AVP neurons), there is a heterogeneity in responsiveness to ghrelin. We therefore carried out additional recordings in which we correlated the electrophysiological responses to the mRNA expressed in individual neurons. We collected the mRNA content from 74 cells, of which 45 (61%) were positive for β-actin mRNA, and were used for this analysis. RT-PCR for AVP, OT, CRH, and TRH was performed on cytoplasm extracted from each neuron following assessment of ghrelin effects using whole cell recording (**Figure 9**). As illustrated in **Figure 10A**, this analysis demonstrated that both ghrelin responsive CRH (3/4 neurons) and TRH (4/5 neurons) expressing neurons demonstrated only hyperpolarizing responses. In contrast, both AVP- (20% depolarized, 40% hyperpolarized, *n* = 35), and OT- (11% depolarized, 44% hyperpolarized, *n* = 9) expressing neurons showed mixed responses to ghrelin, while OT showed a majority of hyperpolarizations. Interestingly, neurons that did not express any of the four tested genes (*n* = 8) showed a higher percentage of depolarizations (50%), with the remainder being either hyperpolarizations or unaffected (25% each).

We also have correlated mRNA expression with our electrophysiological classification of neuroendocrine (**Figure 10B**), pre-autonomic (**Figure 10C**) or magnocellular (**Figure 10D**) phenotypes. These data highlight the unexpected observation that AVP was expressed in the majority of PVN neurons (30/45). Most CRH-expressing neurons co-expressed AVP mRNA (3/4), while the only OT-expressing neuron that depolarized in response to ghrelin presented more AVP than OT mRNA.

## Discussion

The PVN is involved in the control of a series of physiological responses, including the response to osmotic stimuli, metabolic disturbances, cardiovascular and autonomic control, the stress response and secretion of pituitary hormones, and thus, unsurprisingly, this nucleus consists of distinct subpopulations of neurons (Stern, 2015). Ghrelin modulates all the aforementioned physiological responses (Müller et al., 2015), which suggests the PVN as a target for ghrelin actions. Previous studies have assessed the effects of ghrelin on neuronal excitability in the ARC (Cowley et al., 2003; Andrews et al., 2008), hippocampus (Diano et al., 2006; Ribeiro et al., 2014) and area *postrema* (Fry and Ferguson, 2009). However, to date studies examining the effects of ghrelin on the activity of PVN neurons have only reported effects on inhibitory (Cowley et al., 2003) and excitatory (Kola et al., 2008) post-synaptic currents. In this study we show that ghrelin affects the spike frequency of the majority of PVN neurons, either decreasing or increasing their activity. While inhibitory actions of ghrelin were also demonstrated in ARC neurons (Cowley et al., 2003; Yousheng et al., 2008), the fact that ghrelin acts through GHSR, a *G*_q_ coupled receptor that increases intracellular calcium (Cowley et al., 2003), supports the idea that direct actions of ghrelin would be primarily excitatory. Although the majority ghrelin effects are mediated by GHSR, some of ghrelin effects are maintained in GHSR knockout mice (Uchida et al., 2013), which suggests that another receptor may be involved in the responses to ghrelin. In order to evaluate whether the effects were dependent on direct effects on PVN neurons or mediated by previous activation of neurons within the slices, we assessed the effects of ghrelin in the presence of GABAA and glutamate antagonists, and demonstrated that the inhibitory actions of ghrelin were absent when the transmission mediated by these rapid amino acids was blocked. Glutamate induces excitatory responses within the PVN, while GABA is mostly involved in inhibitory responses (Ferguson et al., 2008), suggesting that the different effects in the presence of the antagonists are due to the blockade of GABA actions. These results provide evidence that the direct effects of ghrelin on PVN neurons are excitatory, and that the inhibitory effects are dependent on the excitation of a GABAergic inhibitory circuit. These inhibitory afferents to the PVN may arise from inhibitory interneurons within the PVN or projections from other nuclei present in the slice, such as the ARC. The ARC is activated by ghrelin and sends projections to the PVN, however, some of the effects of ghrelin administration persist in ARC ablated mice, which indicates that the PVN is capable of responding directly to ghrelin (Cabral et al., 2016). Interestingly, both intra-PVN administration of ghrelin (Currie et al., 2005) and the activation of GABAA receptors in the PVN (Tsujii and Bray, 1991; Pu et al., 1999) increase food intake, and immunofluorescence studies demonstrated that ghrelin binds to GABA-expressing neurons in the PVN (Cabral et al., 2016). Therefore, we suggest that ghrelin directly activates GABAergic neurons in the PVN, that, in turn, inhibit other PVN neurons.

Our patch clamp recordings also show that ghrelin primarily depolarizes pre-autonomic PVN neurons, effects that presumably underlie some of the previously demonstrated effects of central ghrelin on autonomic output. ICV ghrelin affects sympathetic responses including, inhibition of renal sympathetic nerve activity (Matsumura et al., 2002; Tanida et al., 2007), increased fat mass and storage in white adipose tissue (Theander-Carrillo et al., 2006), and inhibition of fat oxidation in brown adipose tissue (Yasuda et al., 2003; Theander-Carrillo et al., 2006). Indeed, intra-PVN administration of ghrelin showed similar sympathetic-dependent inhibition of the brown-adipose tissue (Mano-Otagiri et al., 2009), and increases FOS in the nucleus of the solitary tract (Olszewski et al., 2003). Additionally, non-secretory PVN neurons may participate in ghrelin effects on other physiological functions such as anxiety-like behavior (Currie et al., 2012; Wauson et al., 2015) and feeding (Wren et al., 2001; Melis et al., 2002; Olszewski et al., 2003; Shrestha et al., 2004) which are increased by intra-PVN administration of ghrelin.

Magnocellular and neuroendocrine neurons showed mixed responses to ghrelin, thus we attempted to further clarify the effects of ghrelin on magnocellular and neuroendocrine neurons using techniques to describe mRNA expression of AVP, OT, CRH, and TRH; all of which have been previously implicated in the responses to ghrelin (Müller et al., 2015), and correlate this with the effects of ghrelin on individual neurons. We have previously shown that the single cell mRNA expression closely relates to the protein expressed by immunofluorescence, and that the single cell mRNA analysis is a reliable technique to assess the neuronal phenotype (da Silva et al., 2015). Most notably, ICV administration of OT (Olson et al., 1991), CRH (Wang et al., 2007), and TRH (Steward et al., 2003; Schuhler et al., 2007) decreases food intake, and the majority of PVN neurons expressing these neurotransmitters were hyperpolarized in response to ghrelin, supporting the conclusion that this peptide inhibits PVN neurons that express anorexigenic neurotransmitters. Previous studies stated that in the hungry animal the ARC-PVN neurons that inhibit anorexigenic CRH, TRH, and OT neurons are activated, thus inducing food intake (Valassi et al., 2008).

Paraventricular nucleus TRH neurons control TSH and T3/T4 release, thus controlling thyroid function (Pekary and Sattin, 2012). Although the effects of ghrelin on the release of TRH have not been directly demonstrated, negative caloric balance decreases the release of TRH in the median eminence (Joseph-Bravo et al., 2015). Conversely, leptin – an anorexigenic hormone that opposes ghrelin actions – increases TRH release (Guo et al., 2004; Ghamari-Langroudi et al., 2010). This indicates that in the hungry animal the TRH axis is inhibited, thus decreasing T3 and T4 release and reducing energy expenditure. PVN CRH neurons also induce thermogenesis (Richard et al., 2002). Ghrelin is known to reduce energy expenditure (Tschop et al., 2000; Theander-Carrillo et al., 2006), possibly the inhibition of TRH and CRH neurons in the PVN contributes to this response.

Intra-cerebro ventricular ghrelin increases FOS expression on OT neurons in the PVN (Olszewski et al., 2007), and ghrelin induces OT release in hypothalamic cell culture (Gálfi et al., 2016). The release of OT in response to ghrelin, however, has not been demonstrated *in vivo*. Magnocellular OT neurons were mostly hyperpolarized by ghrelin, which suggests that ghrelin would not increase plasma levels of OT. Additionally, OT is involved in the modulation of other responses such as reproduction, social behavior and feeding (Spetter and Hallschmid, 2017). OT is present in pre-autonomic neurons (Sawchenko and Swanson, 1982), which indicates this peptide is involved in the control of these responses. The present results, together with the observations of effects caused by intra-PVN administration of ghrelin on food intake (Wren et al., 2001; Melis et al., 2002; Olszewski et al., 2003; Shrestha et al., 2004); and effects of ghrelin on neuroendocrine (Wren et al., 2002; Gálfi et al., 2016) and autonomic control (Matsumura et al., 2002; Yasuda et al., 2003; Theander-Carrillo et al., 2006; Tanida et al., 2007) indicate the PVN as a major site of action for ghrelin, therefore contributing to the response to caloric imbalance.

AVP mRNA, was co-expressed with all peptides assessed in this study. The cross-talk between AVP and CRH in the control of the hypothalamic-hypophysial-adrenocortical axis is well established (Keller-Wood, 2015), and AVP is essential for the increased CRH release in response to ghrelin in hypothalamic cell culture (Nemoto et al., 2011), which suggests an interplay between these peptides in ghrelin responses. The co-expression of AVP and OT, was previously shown in single-cell analysis of magnocellular neurons on the supraoptic nucleus (Pires da Silva et al., 2016) and PVN (Hoyda et al., 2009). The unexpected ubiquitous presence of AVP mRNA, indicates that this neurotransmitter is involved in more than AVP secretion to the circulation. AVP integrates neurosecretory and pre-autonomic populations of PVN neurons, through a mechanism deemed “volume transmission,” in which the release of a neuromodulator by dendrites into the extracellular medium influences the activity of juxtaposed neurons that are not anatomically connected by axon-dendrite synapses (Stern, 2015). The dendro-dendritic AVP release by the PVN has been shown to be important for autonomic control (Lozić et al., 2016) and neurosecretory and pre-autonomic PVN neurons have been shown to interact in this manner (Son et al., 2013). Ghrelin influences adiposity and energy utilization (Theander-Carrillo et al., 2006), volume transmission could potentially mediate these effects, since they depend on long-term alteration in neurotransmission/neuromodulation. The influence of ghrelin on volume transmission poses an interesting question to be assessed in future studies. The majority of depolarizations in response to ghrelin were found in neurons that did not express any of the tested genes, effects which are therefore likely to be on other PVN neuronal subgroups such as those expressing other transmitters such as glutamate (Hrabovszky et al., 2005) and GABA (Park et al., 2007), indeed, previous studies demonstrated that icv ghrelin binds to GABA-expressing neurons in the PVN (Cabral et al., 2016). The observations that ghrelin inhibits several neuronal subgroups in the PVN, and that these inhibitions disappear in the presence of GABAA and glutamate antagonists suggest that ghrelin increases the activity of inhibitory neurons within the PVN, in turn decreasing the activity of TRH, CRH, and OT neurons. However, this hypothesis needs further testing, since the number of TRH and CRH expressing neurons obtained through this technique was low.

## Conclusion

Ghrelin affects all types of PVN neurons, possibly increasing the activity of GABAergic neurons that inhibit anorexigenic OT-, CRH-, and TRH-expressing neurons, increasing food intake and reducing energy expenditure. Additionally, the ubiquitous presence of AVP mRNA, suggests a function of this peptide that surpasses that of a classical neurotransmitter, possibly being involved in the integration of the different neuronal populations within the PVN. Further studies are necessary to determine the neural circuitry involved in the PVN participation on the responses to ghrelin, with special attention to the different nuclei to which these neurons send efferent projections; and to the intra-PVN integration of these responses.

## Author Contributions

Rd-S, AF, and AM designed the study. All authors participated in data analyses or acquisition, drafted/revised the manuscript, approved the final version, and agreed to be accountable for all the aspects of the work.

## Conflict of Interest Statement

The authors declare that the research was conducted in the absence of any commercial or financial relationships that could be construed as a potential conflict of interest.
